# Savanna chimpanzee (*Pan troglodytes*) crop feeding at Dindefelo, Senegal: challenges and implications for conservation

**DOI:** 10.1007/s10329-024-01125-9

**Published:** 2024-04-30

**Authors:** Laia Dotras, Amanda Barciela, Manuel Llana, Jordi Galbany, R. Adriana Hernandez-Aguilar

**Affiliations:** 1 Jane Goodall Institute Spain in Senegal, Dindefelo Biological Station, Dindefelo, Senegal; 2 Department of Social Psychology and Quantitative Psychology, Faculty of Psychology, University of Barcelona, Barcelona, Spain; 3 Department of Clinical Psychology and Psychobiology, Faculty of Psychology, University of Barcelona, Barcelona, Spain; 4 Institute of Neurosciences, University of Barcelona, Barcelona, Spain; 5 Serra Hunter Programme, Generalitat de Catalunya, Barcelona, Spain

**Keywords:** Crop foraging, Human-chimpanzee interactions, Human-chimpanzee coexistence, Interspecies aggression, Conflict mitigation

## Abstract

Chimpanzees (*Pan troglodytes*) are categorized as Endangered by the International Union for Conservation of Nature, and habitat loss due to conversion of land for agriculture is one of the major threats to wild populations of this species. This challenging scenario can lead to negative human-chimpanzee interactions, including crop feeding. Chimpanzees consume crops across their geographical range, although little is known about this behavior in savanna habitats. Here we provide new evidence of crop feeding by savanna chimpanzees. We conducted our observations at Dindefelo, a community nature reserve in southeastern Senegal. The chimpanzees were observed to feed on mango (*Mangifera indica*) and also on baobab (*Adansonia digitata*), a wild species considered a crop by local people when found in and around villages. Although local people use the fruits of these species for food and income, they tolerated crop-feeding events until recently. In 2023, a case of harassment of a crop-feeding chimpanzee in a mango orchard was witnessed, and four days later a chimpanzee corpse was found at the same place. We conclude that habitat conversion into agricultural fields, uncontrolled bush fires and extraction of wild fruits are the important factors influencing crop-feeding events at Dindefelo. Our findings highlight the need to better understand human-chimpanzee interactions in the anthropogenic landscape of Dindefelo to help mitigate negative attitudes and behaviors towards chimpanzees.

## Introduction

Habitat loss due to the conversion of natural vegetation into agricultural fields is considered the main threat to non-human primate (hereafter primate) species worldwide (Estrada et al. 2017), and it can also be a source of negative human-primate interactions (Hockings 2016; Hockings and Humle 2009). In anthropogenic landscapes, some primate species living in close proximity to humans, such as baboons (*Papio* spp.), macaques (*Macaca* spp*.*) and savanna monkeys (*Chlorocebus* spp.), are perceived as problematic by farmers because they feed on their crops (Lee and Priston 2005).

In sub-Saharan Africa, habitat loss and fragmentation are increasing due to the rapid expansion of agriculture (Brink and Eva 2008). In West Africa alone, between 1975 and 2013, more than 500,000 km^2^ of land was converted into cultivated areas (Herrmann et al. 2020). The negative human-chimpanzee interactions that can arise as a result of the expansion of agricultural land are considered an important threat to the conservation of the Critically Endangered western chimpanzee (*Pan troglodytes verus*) [International Union for Conservation of Nature (IUCN) 2020]. One type of behavior leading to these negative interactions, crop feeding, has been reported in chimpanzees in several West African countries (Hockings and McLennan 2012), including Guinea (Hockings et al. 2009; Humle 2003), Guinea Bissau (Hockings and Sousa 2013) and Sierra Leone (Garriga et al. 2018).

Chimpanzees consume crop species across their geographical range (reviewed in Hockings and McLennan 2012), but there is little information about this behavior for populations living in savanna habitats. This behavior has only been observed in Senegalese savannas, albeit rarely, specifically in Angafou, Fongoli and Heremakhono (Gaspersic and Pruetz 2011; Lindshield et al. 2017, 2021a; Wessling et al. 2019). To our knowledge, no detailed descriptions of crop feeding exist for savanna chimpanzees.

Here we describe mango (*Mangifera indica*) and baobab (*Adansonia digitata*) feeding by chimpanzees at Dindefelo, a hot and dry anthropogenic savanna habitat in Senegal. At this site, local people use baobab fruits as food and a source of income, as in other areas of Senegal (Gaspersic and Pruetz 2011; Lindshield et al. 2021b), and perceive ownership of baobab trees located in and around villages. Thus, we considered these guarded baobab trees as crops because of their importance for the local population. Fruit from non-guarded baobab trees or mango trees in abandoned orchards were not considered crops. We provide the first description of crop-feeding behavior in savanna chimpanzees. We also report a case of harassment of an adult male chimpanzee that entered a mango orchard next to a village, and the subsequent discovery of a chimpanzee corpse at the place where the harassment had been witnessed a few days earlier.

## Methods

### Study area and subjects

Our study area, Dindefelo, is a 14,000-ha community nature reserve located in the Kedougou region, southeast Senegal, along the Guinean border. The elevation ranges from 150 to 450 m above sea level, and there are steep slopes that reach a lateritic plateau. The habitat is a Sudano-Guinean savanna composed of different vegetation types: woodland, shrubland, bamboo woodland, grassland, and evergreen forests. Agricultural land and some degraded areas are also found there. The climate is highly seasonal, with a long dry season lasting from November to May, and a rainy season from June to October. The mean annual rainfall is 1129 mm and the mean annual temperature is 28.5 ℃. There are 14 villages and hamlets in and around the reserve. Most local people belong to the Peulh and Malinke ethnic groups, but members of other ethnic groups, including the Bassari, Bedik, Djallonke and Coniagui, also live in Dindefelo [Jane Goodall Institute Spain (JGIS) in Senegal and A.P.E.S. Wiki team 2023]. The creation of new fields for the cultivation of crops, collection of wild fruits and non-timber forest products, grazing of livestock, cutting of tree branches to provide fodder for domestic animals, and use of water points for washing and laundry occur in the study area (JGIS and A.P.E.S. Wiki team 2023; Pacheco et al. 2012; Ramon et al. 2017). The highest waterfall in Senegal is located inside the reserve and attracts thousands of national and international tourists annually (Camon et al. 2020). Despite these different forms of human impact, Dindefelo possesses a rich biodiversity, including five other primate species besides chimpanzees: green monkeys (*Chlorocebus sabaeus*), patas monkeys (*Erythrocebus patas*), lesser bushbabies (*Galago senegalensis*), Guinea baboons (*Papio papio*) and Endangered king colobus monkeys (*Colobus polykomos*) (Dotras et al. 2022).

A total of 53 adult chimpanzees belonging to two communities have been identified at Dindefelo, using camera trap footage and direct observations (JGIS unpublished data). The main threat for chimpanzees in the reserve is habitat loss due to land conversion for subsistence agriculture and uncontrolled bush fires (JGIS and A.P.E.S. Wiki team 2023; Pacheco et al. 2012). The JGIS carries out research and conservation programs to protect the chimpanzees and their habitat and to foster sustainable development of the local human communities. Non-provisioned habituation of chimpanzees in the reserve for research and ecotourism was started in 2009, which resulted in a few individuals becoming partly habituated, but most chimpanzees in the reserve remain unhabituated. Since 2018, only indirect, non-invasive techniques have been used to gather data on chimpanzee behavioral ecology and health.

**Fig. 1:**
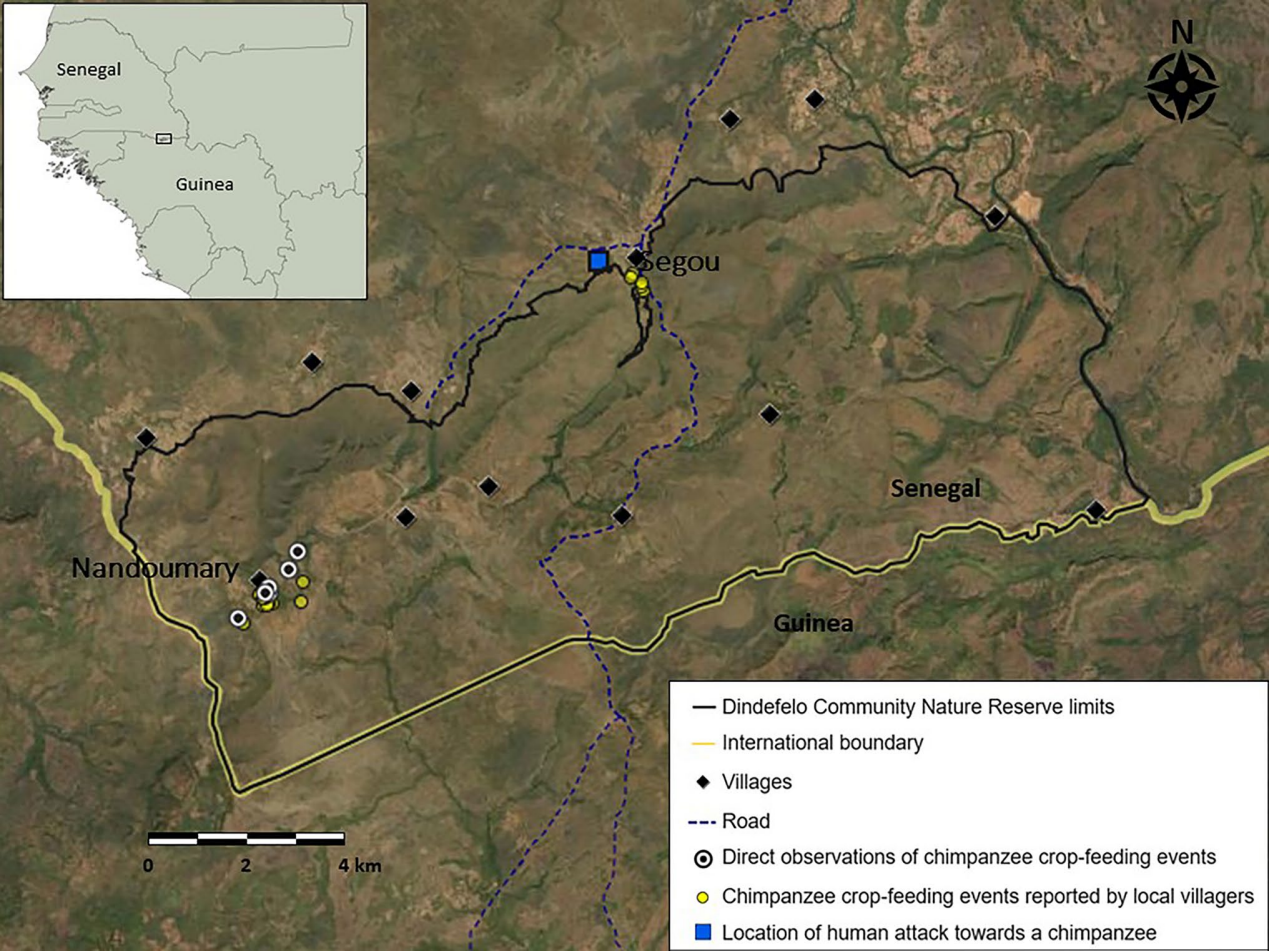
Map of the study area showing the locations of direct observations and local villagers' reports of chimpanzee crop-feeding events, and the location where a case of harassment of a chimpanzee took place

Our observations took place in two villages in the reserve: Nandoumary (12º20′36.05″N, 12º21′27.55″W) and Segou (12°24′36.82″N, 12°16′59.14″W) (Fig. 1). Each of these two villages falls within the range of one of the two chimpanzee communities. In Segou, after written permission had been obtained from farmers, two camera traps (a Bushnell Core DS 30 MP and a Bushnell NatureView HD Essential) were installed in mango orchards where chimpanzees had been observed to crop feed the previous year. Both cameras were set up to record 1-min-long videos with a 1-s delay. They were deployed from February to March 2023, after which they were removed when all the fruits had been harvested for commercial exploitation. In Nandoumary, permission was not given by farmers and thus no cameras could be installed.

## Results

Sporadic events of chimpanzees entering Nandoumary and Segou to forage on mango and baobab fruits have been reported by local villagers since 2015 and 2018, respectively. However, it was not until 2021 that we recorded crop-feeding events in the reserve.

### Nandoumary village

**Fig. 2a–d:**
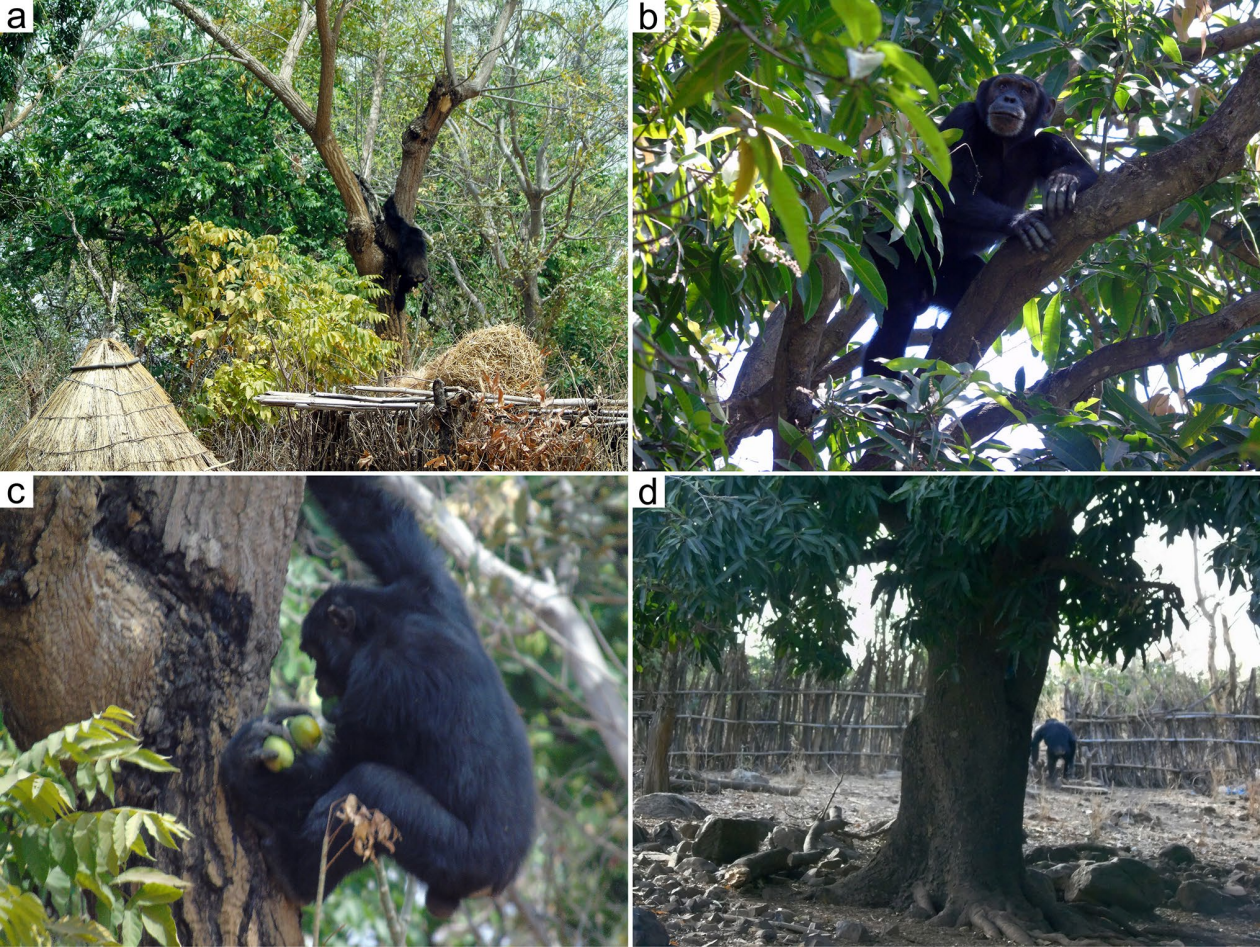
Crop feeding by chimpanzees in Nandoumary village, Dindefelo. **a-c** Adult male carrying mango fruits next to a house. **b** Adult male on a mango tree located in the village. **d** Adult male leaving the compound of a house after accessing mango trees

Between February and April 2021, we recorded seven direct observations of chimpanzees in Nandoumary village foraging on and eating fruits from mango trees. Four of these trees were in household gardens and three were directly adjacent to the village, within 40 m from the outer fence. The chimpanzees ate the fruits in the mango trees or carried them to the forest (Fig. 2). The size of parties in these events ranged from one to three unidentified adult males. On one occasion, 12 individuals were observed approaching a mango orchard, but only two actually collected and consumed fruits. During the same period, Nandoumary villagers reported an additional 19 chimpanzee visits to the village to collect this domestic fruit (Fig. 1). In addition to mangoes, we observed chimpanzees on two separate occasions collecting and eating baobab fruits from trees located inside the village. Chimpanzees regularly cross the village to reach forest patches to collect wild fruits, such as *Saba senegalensis*, *Diospyros mespiliformis* and* Cordyla pinnata*.

During direct observations, even when the chimpanzees passed through Nandoumary village and walked next to the houses, the local people behaved calmly, neither approaching nor trying to scare the chimpanzees away. On one occasion, a small group of children was playing in a mango orchard at the edge of the village when a group of three chimpanzees entered the orchard; the children became scared and ran into the village. Multiple oral reports indicate that this is a consistent behavior of children when they encounter chimpanzees. The crop-eating chimpanzees seemed to have no particular fear of people, as they either remained at a site despite the presence of humans, or quietly returned to the forest when they encountered them.

### Segou village

Since 2018, there have been occasional reports by local people from Segou of chimpanzees visiting mango trees located within the village. During the entire mango fruiting season of 2023 (February–May), the villagers reported seven visits of chimpanzees to mango trees (Fig. 1), but no images of chimpanzees were obtained in any of the 726 videos recorded by the two installed camera traps.

### Case of a crop-feeding chimpanzee harassed by people in Segou village

On 13 March 2023, a JGIS field assistant went to investigate reports by a local farmer of the presence of a chimpanzee in a mango orchard close to Segou village. The field assistant found two 15-year-old boys, with six barking dogs, throwing stones at an adult male chimpanzee who was at the top of a fig tree (*Ficus ingens*) after having been chased from a nearby mango tree. The JGIS field assistant informed the boys that attacking a chimpanzee was dangerous and forbidden by law, and asked them to return to the village. The chimpanzee appeared to be very frightened and was hiding in the highest part of the tree, which prevented the assistant from photographing him to enable his identification. After chasing the dogs away, the field assistant left the site to allow the chimpanzee to leave.

**Fig. 3a, b:**
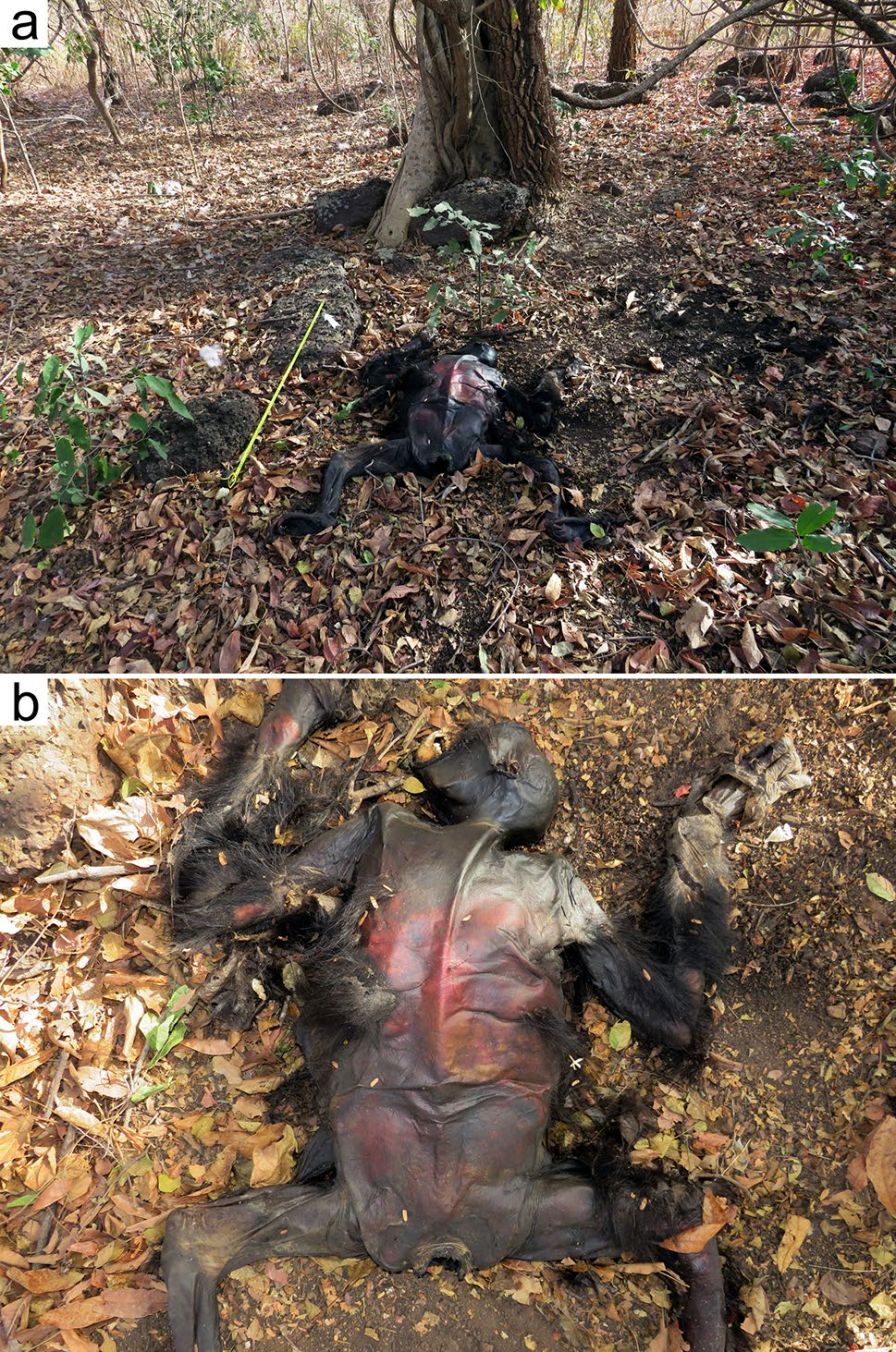
Adult chimpanzee corpse found in Segou village at the same location where a chimpanzee had been harassed by humans four days before, on 13 March 2023

On 17 March 2023, we were informed of the presence of a dead chimpanzee in the same orchard by a villager. After reporting the case to the local authorities, we went to examine the corpse and collect biological samples. The body was in a prone position, under the same tree where the JGIS field assistant had last seen the chimpanzee who was being harassed four days earlier. This location was 172 m north of the reserve’s northern limit (Fig. 1). We were unable to identify the individual or determine its sex. Neither could we determine nor reasonably speculate about the cause of death. The chimpanzee appeared to be a young adult, as all its teeth were present and showed little wear. No obvious cuts or wounds were visible on its limbs or head, but there were bruises on its back. The body appeared to have been dragged along the ground for approximately 1 m (Fig. 3; note that the images contain graphic content). After obtaining hair and tissue samples from the corpse, it was buried in accordance with safety guidelines and a skeletal material recovery protocol (Garrod et al. 2015).

## Discussion

In February 2021, we directly observed chimpanzee crop feeding at Dindefelo for the first time. However, according to information obtained from villagers, this behavior had occurred before. Dindefelo chimpanzees only target mango and baobab fruits, and we have no evidence that they consume other types of crops present in the reserve that are exploited by conspecifics in other sites across Africa, such as maize (*Zea mays*), orange (*Citrus sinensis*) or papaya (*Carica papaya*) (Hockings and McLennan 2012). Local farmers at Dindefelo regularly mention that chimpanzees cause no harm to non-fruit crops there, although these are intensively guarded, normally by children making noise and carrying sticks, which may dissuade chimpanzees from visiting them.

At Dindefelo, humans and chimpanzees have been peacefully coexisting due to a cultural taboo of the Peulh ethnic group against the killing and eating of chimpanzees (Ham 1998). When a surprise encounter between humans and chimpanzees occurs, the former either leave the site or behave calmly. Farmers tolerate chimpanzees when they occasionally access guarded mango and baobab trees, as they consider that the apes’ presence prevents other, more destructive primates, such as baboons and green monkeys, from damaging their trees. This is also the case even in mango orchards belonging to impoverished local families. Mangoes provide food and are also a cash crop, providing a source of income during the dry season, when farmers cannot depend on rain-fed crops. In fact, mango has been identified as a potentially widespread source of negative human-chimpanzee interactions across the entire chimpanzee range (Hockings and McLennan 2012). Other potential sources of negative human-chimpanzee interactions at Dindefelo include artificial beehives and water points during the late dry season (JGIS unpublished data; Pacheco et al. 2012).

Increasing habitat loss due to the conversion of natural areas into agricultural fields and uncontrolled, late dry season bushfires decrease chimpanzee food availability and could potentially explain the apparent rising number of crop-feeding events at Dindefelo. An additional factor may be the increase in wild fruit extraction by humans in the reserve (JGIS unpublished data). Competition for wild fruits (e.g. *S. senegalensis*, *A. digitata* and *Tamarindus indica*) between savanna chimpanzees and humans occurs in Senegal (Gaspersic and Pruetz 2011; Lindshield et al. 2017, 2021b; Pruetz 2002; Ramon et al. 2017), and the National Statistics and Demography Agency of Senegal recently reported an increase in the extraction of wild fruits by humans in the region where chimpanzees exist in the country (Agence Nationale de la Statistique et de la Démographie 2021). However, in Bulindi in Uganda and Bossou in Guinea, seasonal crops, such as mangoes, are consumed by chimpanzees when available, independently of wild fruit availability (Hockings et al. 2009; McLennan 2013).

The increasing exposure of chimpanzees to people and their activities in recent decades could be a factor that has led to a reduction in their fear of humans. The significant presence of tourists visiting the Dindefelo waterfall (JGIS and A.P.E.S. Wiki team 2023) and the years when habituation efforts were undertaken could also have contributed to this decrease in the chimpanzees’ fear of humans. However, in Fongolimbi, a region separated from Dindefelo by the Gambia river, no habituation or tourism have ever taken place, yet local people report that chimpanzees feed on mango in some villages of this region.

If the number of crop-feeding events continues to increase at Dindefelo, a reduction in the locals’ tolerance of chimpanzees could occur, as has been observed in Bossou (Hockings et al. 2012), and human harassment of chimpanzees, such as the single case we report here, could become more common. This could increase the probability of people being injured by apes, especially if the latter are provoked (Hockings et al. 2010). As a response to the case of harassment that we recorded, we have organized awareness-raising activities in villages and schools located in the reserve to inform people about how to behave if they encounter a chimpanzee, and to acquaint them with the legal protection of this Critically Endangered subspecies.

Chimpanzees at Dindefelo, like all savanna populations of chimpanzees in West Africa, face extreme conditions because of the dry, hot, and highly seasonal environment they inhabit (Lindshield et al. 2021a). The impacts of these extreme conditions are exacerbated by the increase in human activities that we are documenting in the reserve (JGIS and A.P.E.S. Wiki team 2023). Understanding the underlying causes of chimpanzee crop feeding at Dindefelo, and gathering periodic information on local people’s perception of chimpanzees, can help towards reaching a more sustainable coexistence between the two species, while also taking into consideration the needs of the local human population. As all chimpanzees in Senegal inhabit savannas, and most of them live in unprotected areas where the human population is increasing (Ndiaye et al. 2018), crop feeding may become more common in chimpanzees in the country.
